# Identification, utilisation and mapping of novel transcriptome-based markers from blackcurrant (*Ribes nigrum*)

**DOI:** 10.1186/1471-2229-11-147

**Published:** 2011-10-28

**Authors:** Joanne R Russell, Micha Bayer, Clare Booth, Linda Cardle, Christine A Hackett, Pete E Hedley, Linzi Jorgensen, Jenny A Morris, Rex M Brennan

**Affiliations:** 1 Cell & Molecular Sciences, James Hutton Institute, Invergowrie, Dundee DD2 5DA, UK; 2 Biomathematics and Statistics Scotland, James Hutton Institute, Invergowrie, Dundee DD2 5DA, UK

## Abstract

**Background:**

Deep-level second generation sequencing (2GS) technologies are now being applied to non-model species as a viable and favourable alternative to Sanger sequencing. Large-scale SNP discovery was undertaken in blackcurrant (*Ribes nigrum *L.) using transcriptome-based 2GS 454 sequencing on the parental genotypes of a reference mapping population, to generate large numbers of novel markers for the construction of a high-density linkage map.

**Results:**

Over 700,000 reads were produced, from which a total of 7,000 SNPs were found. A subset of polymorphic SNPs was selected to develop a 384-SNP OPA assay using the Illumina BeadXpress platform. Additionally, the data enabled identification of 3,000 novel EST-SSRs. The selected SNPs and SSRs were validated across diverse *Ribes *germplasm, including mapping populations and other selected *Ribes *species.

SNP-based maps were developed from two blackcurrant mapping populations, incorporating 48% and 27% of assayed SNPs respectively. A relatively high proportion of visually monomorphic SNPs were investigated further by quantitative trait mapping of theta score outputs from BeadStudio analysis, and this enabled additional SNPs to be placed on the two maps.

**Conclusions:**

The use of 2GS technology for the development of markers is superior to previously described methods, in both numbers of markers and biological informativeness of those markers. Whilst the numbers of reads and assembled contigs were comparable to similar sized studies of other non-model species, here a high proportion of novel genes were discovered across a wide range of putative function and localisation. The potential utility of markers developed using the 2GS approach in downstream breeding applications is discussed.

## Background

In many species the main limitation to understanding and characterising important traits is the lack of sufficient genetic markers for the development of high-density genetic maps and association studies. Large numbers of markers, such as Simple Sequence Repeats (SSRs) and Single Nucleotide Polymorphisms (SNPs), are required to assist in identifying genes that underlie genetic variation. For many crop and horticultural species, genetic linkage maps have now been developed and Quantitative Trait Loci (QTL) have been assigned to large chromosomal regions, but so far candidate genes have been identified for only a few of these [1]. The need for more genetic markers is recognised and until recently has been a major challenge and expense. With the introduction of new sequencing technologies, traditional low-throughput methods of marker development have been superseded [2]. These technologies are often referred to as 'Second Generation Sequencing' (2GS) and the platforms include the Illumina Genome Analyzer, the Roche 454 FLX and the Applied Biosystems SOLiD systems, all of which are widely used for shotgun genome sequencing and SNP discovery [3-9].

Deep-level 2GS technologies are now being applied to non-model species as a viable and favourable alternative to Sanger sequencing, despite the absence of a reference genomic sequence on which to map the short reads. Expressed Sequence Tags (ESTs), derived from the RNA-based transcriptome, have been extremely useful resources to assist marker development [10] and, by utilising 2GS technologies, transcripts can be sequenced to a greater depth, enabling discovery of novel gene sequences at a fraction of the cost and time taken previously. This approach is particularly useful in species where there is little genome information, allowing a large number of SNPs to be identified from across a wide range of transcripts [11]. Recently, several such studies based on high-throughput transcriptome sequencing have been carried out in non-model plant species, including maize, grapevine, eucalyptus, olive and common bean [3,6,4,7,12].

Blackcurrant (*Ribes nigrum *L.) is taxonomically isolated within the Saxifragaceae and current genomics resources are extremely limited. As with many economically important woody perennial species, breeding of *Ribes *is a long-term process due to the highly heterozygous germplasm available and the long generation time, so there is an obvious incentive to develop marker-assisted breeding strategies to reduce the timescale for selection of superior genotypes. Previously, we have constructed cDNA libraries from developing fruit and buds, and Sanger-sequenced several thousand ESTs [13,14]. From these libraries, forty-three SSR and sixteen SNP markers have been mapped genetically and, together with AFLPs, a number of markers associated with key phenological and fruit quality traits identified. Despite these being relatively large sequencing efforts at the time, we were still only able to generate a sparsely populated framework map of 538 cM with QTL spanning 5 to 10 cM. 2GS technologies now offer the opportunity to generate large numbers of novel markers from which to construct high-density genetic linkage maps.

The aim of our current study was to perform large-scale SNP discovery from gene coding regions of blackcurrant using 2GS 454 pyrosequencing. Once SNPs were identified, an efficient means of genotyping was required. Previous studies have validated only a small proportion of the identified SNPs, usually by Sanger re-sequencing [4,15]. High-density assays for SNP detection have recently been developed and one such platform from Illumina enables simultaneous assays of 384 markers from a single DNA sample. A subset of polymorphic SNPs from blackcurrant, representing a diverse set of genes, was therefore used to develop a 384 SNP Oligo Pool All (OPA) assay on the Illumina BeadXpress platform. In addition, 2GS transcriptome sequencing facilitated identification of novel EST-SSRs which are proven robust marker types [10,16,17]. To facilitate validation of these SNPs and SSRs, two segregating mapping populations and a diverse set of germplasm, 480 samples in total, were assayed.

## Results

The overall objective of this study was to determine whether 2GS technology would enable significant gene discovery in *Ribes nigrum *and whether these short reads could be assembled *de novo *for efficient isolation and development of novel genetic markers. In this study, over 700,000 sequence reads generated from cDNA derived from developing blackcurrant buds of parental genotypes gave sufficient coverage to detect *c*. 7,000 SNPs, a subset of which were validated via the Illumina BeadXpress genotyping platform.

### Transcriptome sequencing, contig assembly and gene annotation

A total of 712,814 high-quality sequence reads derived from pooled RNA extracted from developing buds of each of the *Ribes *parents S10 (226,248 reads) and S36 (485,566 reads) were screened for adaptor sequence contamination, leaving 225,334 reads (S10) and 482,959 reads (S36), followed by removal of ribosomal matches, leaving 212,104 reads (S10) and 314,189 reads (S36). We found significantly higher levels of rRNA-derived contamination in S36 (35%) compared to S10 (6%), which was believed to be due to processing-related factors, therefore a further run of S36 was necessary to boost filtered read levels from this parent. The mean read length of the final sets were 214 nt (S10) and 230 nt (S36) respectively. These were subsequently assembled *de novo*, resulting in 33,518 contiguous sequences (contigs) and 12,893 singletons, with a mean contig length of 407 nt (range of 40 nt to 8,440 nt). These contigs and singleton sequences were annotated with descriptors of their closest homologues by running BLASTX searches against the non-redundant protein sequences from NCBI and the peptide models for *Arabidopsis thaliana *from TAIR [18,19], matching 21,527 and 17,280 peptides respectively. The percentage of assembly products scoring significant BLAST hits (i.e. with an e-value of less than 10^-10^) was 52% and 64% respectively, reflecting the high level of novel gene identification for *Ribes *in this study. The BLAST hits resulting from the search against the *Arabidopsis *peptides were also processed further by extracting Gene Ontology (GO) terms for each hit using the GO annotation provided by TAIR (Additional File 1: Figure S1). There was representation of transcripts in all but one of the major GO categories for biological processes, the exception being the "other physiological processes" category. In addition to annotating the assembled contigs, we also compared them with the set of existing Sanger sequenced ESTs from the cultivar Ben Hope (3,327 in total) [20], using the 454 contigs as query sequences in a BLAST search against the Sanger ESTs. A total of 2,688 of the existing Sanger EST contigs were represented in the output from the 454 runs, leaving 639 (19%) without representation, reflecting the difference in tissue provenance between samples.

### Marker development: Single Nucleotide Polymorphisms and Simple Sequence Repeats

A set of 7,245 high-confidence (p > 0.9) *Ribes *SNPs were discovered using GigaBayes software. Parental genotypes were also defined and for the majority of cases, either one parent (4,239 out of 7,245) or both parents (2,684) were heterozygous, and only a small proportion (202) was found where both parents were homozygous. There were only 120 cases where all the reads in the contig originated from the same parent, and these were not considered for further use in this study. As well as SNPs, many of the EST sequences contained repeat motifs. Using Sputnik software [21], 3,179 SSRs were identified, of which over half were trinucleotide, a third dinucleotide, and a small number were tetra- and pentanucleotide repeats.

The 384 SNP assay was designed using Illumina technical support (techsupport@illumina.com). As described in the Methods section, the Illumina SNP selection was based on an absence of neighbouring polymorphisms, repetitive elements or palindromes, which are known to have an adverse effect on success of assays.

### Preliminary analysis of SNPs in the mapping populations

From the 384 SNPs scored, 189 were identified as segregating in mapping population SCRI 9328 using the BeadStudio software (version 3.1). Of these, 75 were heterozygous in the seed parent only, 63 were heterozygous in the pollen parent only and 51 were heterozygous in both parents. Inspection of segregation ratios of the individual markers showed four lines in the population with unexpected genotypes for many SNPs, and these were excluded from subsequent analysis. A cluster analysis of the remaining progeny based on the markers that were heterozygous for the seed parent only showed no particular groupings, but a cluster analysis based on the markers heterozygous for the pollen parent showed a distinct cluster of 46 offspring, none of which had inherited any of the alleles specific to the pollen parent. A chi-squared test was used to compare the segregation ratio of these 46 offspring with the remaining 261 offspring for the markers heterozygous for the seed parent. This found that the segregation ratios were significantly different (p < 0.001) for 72 of the 75 markers, with a segregation ratio close to 1:2:1 for these 46 offspring, but 1:1 for the remaining offspring. These results are consistent with these 46 offspring being selfs and these were excluded from the linkage analysis.

In the MP7 population, 118 of the 384 SNPs were found to segregate using the BeadStudio software. Of these, 50 were heterozygous in cv. Ben Finlay (seed parent) only, 35 were heterozygous in cv. Hedda (pollen parent) only and 33 were heterozygous in both parents. A cluster analysis of the MP7 population showed three lines in the population with unexpected genotypes for many SNPs and these were excluded from subsequent analysis. Cluster analysis showed no evidence for any selfing or other grouping of individuals within this population.

### Linkage analysis of SCRI 9328

**Figure 1:**
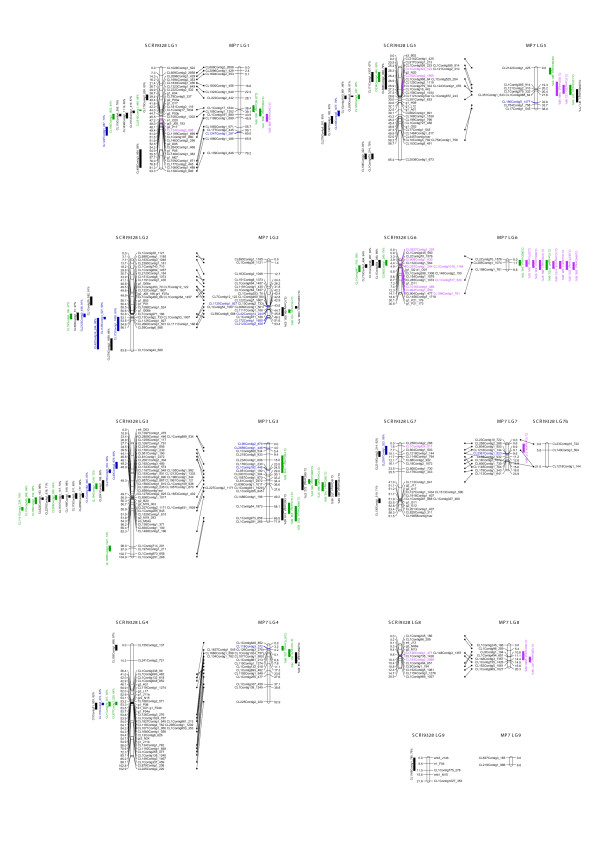
**Linkage maps of the SCRI 9328 and MP7 populations**. with one-lod confidence intervals for the SNP theta scores with R^2 ^> 50%. Different colours show shared QTLs (green), QTLs in SCRI 9328 and markers in MP7 (blue) and QTLs in MP7 and markers in SCRI 9328 (pink).

Both SNP and SSR markers were used in the linkage analysis. No markers were isolated from this population: all were linked with a lod of at least 11 to one or more other markers. Two linkage groups formed at a lod score of three, but the remaining markers only separated at a higher lod, between 7 and 16. This gave ten linkage groups, of which two were small, while the remaining groups had 14-46 markers. The markers within each linkage group were ordered together, rather than separating the markers from the two parents as is sometimes necessary for this type of cross. The fit of the linkage map was, in the authors' experience, unusually good for an outbreeding species. Only five markers were omitted as causing problems with the fit, and JoinMap's mean chi-squared criterion for the resulting maps was below 2.5 for each of the eight large linkage groups. Figure 1 shows the linkage maps, produced using the Mapchart 2.1 software [22]. The linkage groups have the same numbering as in [14], using the SSR markers for identification: the order of the SSR markers shows good agreement with the smaller population. The total map length is 605 cM.

### Linkage analysis of MP7

In this population, six SNP markers were excluded as having highly distorted ratios (p < 0.001). Five markers were isolated at a lod of 4. The remaining markers formed 9 linkage groups using a lod threshold between 5 and 7. There were two small groups, of two and three markers, and seven larger ones of 8-21 markers. Two markers were excluded as causing problems with the fit. The remaining fits were good, again with all mean chi-squared criteria below 2.5. Figure 1 shows the linkage maps, with lines connecting markers to the corresponding ones on SCRI 9328. These show good agreement between the maps. The total map length is 355 cM.

### Analysis of heterogeneity between recombination frequencies

Where there are pairs of SNPs in common between the corresponding linkage groups, the recombination frequencies can be tested for heterogeneity using a chi-squared test implemented in JoinMap 3. A total of 360 pairs of SNPs were examined. Of these, there was no significant heterogeneity (p > 0.05) for 339 pairs, while 15 pairs had significance between 0.05 and 0.01, i.e. a similar number to that expected by chance. Six pairs showed more significant heterogeneity, two pairs on LG7 both involving CL113Contig1_641 were significant with p < 0.005, while four pairs on LG5, all involving CL754Contig1_758, were significant with p < 0.001. Heterogeneity of recombination frequencies is therefore not a widespread problem between these two crosses.

### QTL analysis of the SNP theta scores for the SCRI 9328 population

Inspection of the 384 SNP theta scores for the SCRI 9328 population showed that 15 SNPs had more than 100 missing values. These were excluded from further analysis, leaving 369 SNPs with at most 15 missing values. The range was also examined: the ideal SNP will have a range of one, i.e. a theta score of one for the BB genotype and zero for the AA genotype. SNPs with a range less than 0.05 were excluded from the QTL analysis, leaving a total of 310 SNPs for which the theta scores were mapped. These consisted of 184 SNPs that were mapped as clear bi-allelic markers, five SNPs that segregated as bi-allelic markers but were excluded from the linkage map and 121 SNPs that were considered as non-segregating by BeadStudio.

All 184 SNPs that could be mapped as markers mapped to the same location when their theta scores were used for QTL mapping. Regression of the theta values on the most significant marker explained 71-99% of the variance in the theta values, with a lower quartile of 97%. The five SNP markers that were dropped from the linkage analysis due to their poor fits to the linkage group all mapped to the same groups when the theta scores were analysed as QTL, with regression on the closest marker explaining 90-99% of the variance of the theta score. Two of these markers were heterozygous in both parents, and mapped to a region on LG2 with some segregation distortion. The other three were heterozygous in one parent but, when mapped as QTL, showed associations to the alleles from the other parent.

The 121 remaining SNPs, when mapped as QTL, showed marker associations with the maximum percentage variance explained ranging from 0.7% (i.e. no significant association) to 99%. Thirty-one of the SNPs had a maximum percentage variance of at least 70%, comparable to the SNPs that were also mapped as markers. Significance thresholds for the presence of QTL were established by means of a permutation test [23], using 100 permutations for each of three traits with different ranges, indicating that the maximum percentage variance explained for any of these permuted traits was 6.3%. Thirty-six SNPs had a maximum percentage variance below 6.3% and these will be categorised as without significant QTL. However we are interested here in SNPs where there is substantial, rather than just statistically significant, genetic variance and we have therefore chosen to focus on SNPs where the maximum percentage variance explained by marker regression is greater than 50%. Fifty-two of the 121 SNPs fall in this range. One-lod confidence intervals for these SNPs, together with the five that were a poor fit in the linkage analysis, are shown in Figure 1.

### QTL analysis of the SNP theta scores for the MP7 population

In this population, 251 SNPs had theta scores with a range greater than or equal to 0.05 and at most 10 missing values. One hundred and eighteen of these were scored as markers, with 105 placed on the linkage map. Of the 133 remaining SNPs, 36 mapped as QTL with more than 50% of the variance explained and these are shown in Figure 1. There is good agreement between the positions of the SNP markers in the two populations, whether mapped as markers or as QTL: 15 SNPs mapped as QTL to similar positions on the same chromosome in both populations, 24 SNPs mapped as a QTL in one population and as a marker to a similar position on the same chromosome. Some only mapped in one population. Only one clear discrepancy was found, CL2395Contig1_181. This mapped as a marker in SCRI 9328 to linkage group LG2. As a QTL, it mapped to the same location with 82% of the trait variance explained, but showed smaller, though significant (p < 0.001) peaks on LG3 and LG5. CL2395Contig1_181 did not map as a marker in MP7 but mapped as a QTL to LG5, with 71% of the trait variance explained.

### Validation of SNPs via diversity analysis

The 384 SNPs were also used to examine diversity in a range of 66 *Ribes nigrum *cultivars and 5 related species. The number of polymorphic SNPs was similar to that observed in the original mapping population (207 SNPs *cf*. 190 SNPs). Diversity values for each SNP, measured using Nei's unbiased expected heterozygosity, ranged from 0.030 to the maximum value of 0.500, with an overall mean value of 0.307 (Table 1). The observed and expected heterozygosity values were similar, with a mean inbreeding coefficient of -0.069 (Table 1). Only 22 loci exhibited a minimum allele frequency (MAF) less than 0.050 and 47 with a MAF less than 0.100. Almost half of those scored were shown to be monomorphic in the 5 related species.

**Table 1:** Summary diversity statistics calculated for 207 polymorphic SNPs for 71 *Ribes *germplasm accessions and related wild species.

	Sample Size	Observed Heterozygosity	Expected Heterozygosity	Unbiased Expected Heterozygosity	Fixation Index
Breeding lines	33	0.366	0.333	0.338	-0.090
'Ben' cvs	15	0.374	0.313	0.324	-0.161
Other cultivars	18	0.334	0.307	0.316	-0.072
Wilds	5	0.149	0.217	0.248	0.229
					
Overall Mean		0.306	0.292	0.307	-0.047

'Ben' relates to the series of cultivars released from the breeding programme at JHI.

### Validation of SSRs via mapping and diversity analysis

A subsample of 40 SSRs representing different motif types and repeat numbers were tested using the SCRI 9328 mapping parents and a range of blackcurrant germplasm and related species, gooseberry (*R. grossularia *L.) and redcurrant (*R. rubrum *L). Of the 40 SSR primers designed, 36 amplified in all genotypes tested and of the 10 SSRs which were subsequently fluorescently labeled and visualised using the ABI 3730, 6 were mapped in the segregating population (shown in Figure 1) and 8 were polymorphic in the germplasm collection. The number of alleles ranged from 3 to 8, with a mean value of 2.9 and a mean unbiased expected heterozygosity of 0.397 (Table 2). As with SNP analysis, SSRs showed similar values for observed and expected heterozygosity and a comparable inbreeding coefficient of 0.128 (Table 2). Comparing cultivated and wild accessions, diversity was greater in the wild *Ribes*, although this was associated with high levels of inbreeding (mean F*_IS _*of 0.432 for 5 wild *Ribes*) for all loci, suggesting the presence of null alleles in the wild germplasm.

**Table 2:** Summary diversity statistics calculated for 8 polymorphic SSRs for 68 *Ribes *germplasm accessions and related wild species.

	Sample Size	Mean number of Alleles	Observed Heterozygosity	Expected Heterozygosity	Unbiased Expected Heterozygosity	Fixation Index
Breeding lines	30	3.250	0.346	0.334	0.340	-0.062
'Ben' cvs	15	3.000	0.345	0.368	0.381	0.040
Other cultivars	18	3.875	0.348	0.428	0.440	0.193
Wilds	5	3.500	0.350	0.627	0.701	0.432
						
Overall Mean		2.950	0.303	0.364	0.397	0.128

'Ben' relates to the series of cultivars released from the breeding programme at JHI.

## Discussion

Central to all plant breeding programmes is the identification of genes that control economically important traits. Traditionally this has been achieved by developing genetic maps using a limited number of molecular markers. With the recent advances in sequencing technologies, markers can now be generated on an unprecedented scale [10]. We report the use of 2GS 454 technology to generate over 700,000 reads from cDNA of developing blackcurrant buds, allowing sufficient coverage to identify over 7,000 SNPs and 3,000 SSRs. Below we discuss the attributes of the assembled contigs and singletons and the utility of the SNP and SSR markers to provide an improved genetic map to help identify genes responsible for important traits in blackcurrant.

In terms of read numbers and assembled contigs and singletons, our results were similar to those generated in other 454 transcriptome studies of non-model species [3,4,7,8,15,24]. Of 33,518 contigs and 12,893 singletons, 52% and 64% scored significant BLAST hits to peptide sequences in the public domain, which was higher than that reported for other tree species including *Eucalyptus grandis *(38%) [4] and *Pinus contorta *(32%) [8]. However, these relatively low levels of significant homologies and the presence of ESTs not found in our Sanger EST collection [20] reflect the high proportion of novel genes discovered in this study for blackcurrant. From the peptide homologies and GO annotation analysis (Additional File 1: Figure S1), it was clear that transcripts from a wide range of genes, with respect to putative function and localisation, have been sampled and thereby form the basis of novel gene-specific markers.

Second generation sequencing has been used to identify SNPs in a range of plant species [10]. In this study we identified over 7,000 SNPs from *de novo *assembled blackcurrant EST data. As well as the development of this approach for SNP discovery, we addressed the question of validation and whether *de novo *SNP discovery based upon 2GS data alone can translate into SNP detection assays and, more importantly, useful markers. We designed a multiplex high-throughput SNP detection assay based on the Illumina BeadXpress platform and examined polymorphism across 384 SNPs using two segregating populations and a diverse set of germplasm. Although all SNPs were chosen to be polymorphic from read alignments, we were unable to confirm almost half of putative SNPs from the current assembly by a linkage mapping approach as they did not segregate clearly in the mapping populations. There may be technical reasons why some SNPs do not perform as well as others: Close *et al. *[25] describe some unscorable SNPs due to low GenTrain scores (less than 0.300), even though they had been selected from Sanger sequenced EST collections. Although several of our SNPs fall into this class (13%), the majority of those unconfirmed SNPs appeared in a single cluster with high GenTrain scores and were subsequently scored as monomorphic. These monomorphic SNPs could be sequencing errors masquerading as SNPs or mis-assembled reads, resulting in sequences of gene family members from different regions of the genome being assembled into single contigs. Additional sequencing would be expected to increase the transcriptome space coverage which would ultimately improve the specificity of assembly. Recently, we augmented our blackcurrant ESTs using paired-end Illumina 2GS of the same RNA (data not presented) and found that several of the 454 contigs which led to monomorphic SNPs (~15%) were not supported in the new assembly and that many of the predicted SNPs (~70%) in these contigs also disappeared. This also highlights the recent rapid technical advances in 2GS, in terms of levels of coverage and sequencing fidelity achievable. Indeed, hybrid assemblies derived from multiple 2GS platforms often achieve the most reliable contig datasets. Alternative strategies to RNA-seq include genomic reduction approaches, which aim to reduce gDNA complexity of species with large genomes, such as maize, grain amaranths, common bean and soybean [3,9,12,26-28]. These approaches may suffer less from mis-assembly, by including unique non-coding sequences, however such non-genic markers cannot often be directly related to functionality. As well as reducing the initial complexity, improvements in *de novo *assembly and SNP identification pipelines have recently been developed [29,30].

Using the available analysis software (Illumina BeadStudio v3.1), we were able to map 184 SNPs (48% of assayed SNPs) and 105 SNPs (27% of assayed SNPs) from two blackcurrant mapping populations, SCRI 9328 and MP7 respectively. Although these levels appear relatively low, considering both parents of 9328 were used in the SNP discovery pipeline, other studies which have used mapping parents in the same manner (discovery, detection and subsequent mapping) found similar numbers of SNPs placed on the genetic maps in maize (63%) [27] and in two mapping populations of potato (43% and 48%) [30]. There was good agreement of markers between maps with very little heterogeneity of recombination frequencies. Although these SNPs greatly improved our previous maps, we investigated the monomorphic markers further by mapping the theta score outputs from the BeadStudio analysis as quantitative traits. As these scores are expected to be from a single genetic locus, plus some measurement error, we used a very high threshold of 50% of the trait variance explained by a single position. At this threshold we were able to place 52 of the visually monomorphic SNPs on the SCRI 9328 map and 36 on the MP7 map. In general there was good agreement between positions in the two populations, whether SNPs were mapped as QTL in both populations or as a QTL in one population and a marker in the other. Further SNPs could be mapped as QTL by lowering the threshold. We plan to investigate further how SNP theta scores can best be used in such analyses.

The 384 SNP assay was also used to genotype a set of diverse blackcurrant accessions, including breeding lines, and related cultivated and wild *Ribes *species. Over half of the SNPs were polymorphic with a mean MAF of 0.253, similar to that observed in chicken (0.280) and pigs (0.274) using SNPs from reduced representation libraries [31,11]. Mammadov *et al. *[27] used MAF as a means of measuring polymorphism for SNP markers, and in their maize study using 604 mapped SNPs, 80% had a MAF > 0.100. In our study of 209 polymorphic SNPs, over 75% had a MAF > 0.100. The SNP markers also performed well when comparing diversity to other studies (mean H*_E _*of 0.292 for *Ribes *compared to H*_E _*of 0.350 for chicken [31]) and, as expected for blackcurrant, there was no evidence of inbreeding, with very similar values of observed and expected heterozygosity.

As well as SNPs, several studies have used similar approaches to mine for SSRs, for a range of applications including mapping, systematics, population and conservation genetics [8,16,17,32-35]. The numbers of identified SSRs varied across these studies from almost all (97%) sequences with microsatellites (FIASCO enrichment procedure) [17] to several hundred (single lane of transcriptome sequencing) [33], with most studies falling somewhere in between. In this study, we have identified over 3,000 novel blackcurrant EST-SSRs using 454 2GS which will provide sufficient gene-based markers for most applications. Diversity values from our study (H*_E _*0.152 to 0.825) were comparable with others (eg. in juniper, 0.200 to 0.900) [34], although as expected these were slightly lower than in our previous study using genomic SSRs, with values ranging from 0.184 to 0.908 [36]. However, the effort and time required to develop genomic SSRs is far greater and more costly. Furthermore, we observed significant correlation between the genetic distances matrices generated from SNP and SSR data for the same blackcurrant individuals (20 common accessions; r^2 ^= 0.777, data not shown), corroborating the robustness of these markers for a range of applications.

## Conclusions

We have found the use of 2GS technologies for marker development far superior to any previously described methods (supported in [8]), both in terms of the numbers of SNPs and SSRs identified and in the biological informativeness of those markers. The approach is extremely cost-effective for species with unsequenced genomes and would be greatly improved simply by utilising, or using combinations of, the most up-to-date 2GS technologies available. Informatics analysis of such data is still in its infancy, but on-going improvements to assembly and identification will allow simple selection of the most robust and informative markers from any species into a working assay, thereby enhancing the development of marker-assisted breeding strategies. At the present time, such strategies for breeding in *Ribes *are restricted to a single-gene pest resistance trait [37] but, using the findings reported here, the opportunity to extend early selection to include complex traits such as fruit quality and developmental characters offers exciting prospects for future varietal development in blackcurrant.

## Methods

### Plant material

Leaf buds were sampled from four-year old blackcurrant plants grown in the field at Invergowrie, Dundee (latitude 56.45, longitude -3.06) of both parents of the reference mapping population SCRI 9328 in February 2008, immediately prior to dormancy break, i.e. as the buds began to visibly swell. Buds were flash frozen in liquid nitrogen and stored at -80°C.

The SCRI 9328 population consists of 311 F_1 _full-sib progeny from a pseudo-testcross [38] made by hand in an insect-proof glasshouse between two diverse breeding lines from the James Hutton Institute [14]. In addition, a second F_1 _full-sib mapping population with 95 progeny, designated MP7, from a cross between blackcurrant cvs. Ben Finlay and Hedda, was used in the downstream validation of markers.

A range of *Ribes *germplasm, including 33 breeding lines, 15 commercially available cultivars (Bens) and 5 related wild species (Table 1, 2) were used to determine the diversity of both SNP and SSR markers identified in this study.

### Total RNA extraction

Total RNA was extracted from 100 mg of frozen pooled developing bud material using the Plant RNeasy Mini Extraction Kit (RLC buffer, Qiagen) with the addition of RNA isolation aid (Ambion). RNA quality was checked by spectrophotometry and integrity assessed using a Bioanalyzer (Agilent Technologies).

### Genomic DNA isolation

Young leaf material was harvested from field grown plants of two mapping populations (SCRI 9328 and MP7) and 71 *Ribes *germplasm accessions. Total genomic DNA was extracted using either the method described by Milligan [39] or the DNeasy Mini Extraction Kit (Qiagen). DNA quality and quantity were measured using PicoGreen spectrophotometry (Invitrogen).

### 454 sequencing and quality control

Total RNA from developing buds of *Ribes *parents S10 and S36 were submitted separately to the GenePool Service Facility (University of Edinburgh, UK) for standard transcriptome 454 FLX (Roche) RNA-seq sequencing. cDNA was generated using either SMART (Clontech) or MINT (Evrogen) kits as recommended by the manufacturer. Fragmentation and library preparation were performed as recommended (Roche) prior to running samples. All sequence reads have been submitted to EMBL European Nucleotide Archive (ENA: http://www.ebi.ac.uk/ena/). The reads for each parent were screened for the presence of adapter sequences originating from both the cDNA preparation and the 454 experimental procedures. Adapter contamination was masked using CROSS_MATCH (http://www.phrap.org/phredphrapconsed.html), and then trimmed from the reads using custom perl scripts. The matching quality scores for the reads were also removed. Any reads that had adapter contamination in the middle were discarded as possible chimeric sequences. Following adapter trimming, the sequences were screened for the presence of contaminating ribosomal RNA. A small BLAST database containing ribosomal RNA sequences from a variety of plants was constructed from entries using a keyword search of Genbank. The reads were then searched against this database and any that had a match to a ribosomal RNA sequence with an e-value greater than 1e-10 were discarded.

### Sequence assembly

After adapter and ribosomal sequence trimming, the identifiers of each of the sequences were prefixed with the parental name (S10 or S36), and then all 526,293 sequences were assembled using the tgicl suite (http://compbio.dfci.harvard.edu/tgi/software) running on a single CentOS Linux machine with four processors. The assembly parameters used were the same as those 'relaxed' parameters used in the HarvEST assemblies (http://harvest.ucr.edu), namely the CAP3 parameters -p 75 -d 200 -f 250 -h 90. These were sufficiently relaxed so that SNPs would not be separated into different contigs, thereby allowing SNP discovery. During assembly, 19 reads caused slippage error messages from CAP3 and were therefore removed.

### EST annotation

Contigs were annotated with descriptors of their closest homologues using BLAST (with an e-value cut-off of 1e-10) to search them against the non-redundant protein sequences from NCBI and against the peptide models for *Arabidopsis thaliana *[19]. The BLAST hits resulting from the search against the *A. thaliana *peptides were processed further by extracting Gene Ontology (GO) terms for each hit using the annotation file provided by TAIR (ftp://ftp.arabidopsis.org/home/tair/Ontologies/Gene_Ontology/ATH_GO_GOSLIM.txt). The number of occurrences of each GO ID was then recorded, and the GO ID was resolved against the highest order GO categories that were to be visualised (ftp://ftp.arabidopsis.org/home/tair/Ontologies/Gene_Ontology/TAIR_GO_slim_categories.txt).

### SNP determination

Single nucleotide polymorphisms (SNPs) were discovered in the final assembly using the GigaBayes tool from the laboratory of Gabor Marth at Boston College (http://bioinformatics.bc.edu/marthlab/GigaBayes). GigaBayes detects SNPs and indels in assembly files (ace file format) and, depending on parameter settings, can also output parental genotypes. Both the SNP itself and the parental genotypes are associated with a Bayesian probability value which indicates the degree of confidence in the feature. The parameter settings "--CRL 6 --CAL1 3 --CAL2 3 --PSL 0.9 --QRL 0 --QAL 0 --ploidy diploid --sample multiple" were used to find locations at which both the minor and major alleles are present at least three times per assembled sequence. The minimum read base quality value (--QRL) and minimum aggregate allele quality value (--QAL) flags had to be set to a zero threshold because the assembly software used assigns low base quality scores to the consensus sequence at positions where there is a high degree of variability, such as at SNPs [40]. The GigaBayes output and the contig sequences were visualised and selected using the 'Tablet' software package [41] and submitted to Illumina technical support (techsupport@illumina.com) for design of Illumina GoldenGate SNP assays. The Illumina SNP selection is based on an absence of neighbouring polymorphisms (60 bp flanking sequence on each side between SNPs), repetitive elements or palindromes, since these are known to affect the conversion rate of SNPs into working assays [42,43].

### SSR identification and analysis

SSRs were identified from the assembly using the Sputnik program [21] and oligonucleotide primers were designed using Primer 3 [44]. Primer pairs were tested for their ability to amplify SSR loci according to the protocols described in [36]. SSR loci were visualised using ABI PRISM^® ^3730 Genetic Analyzer and alleles scored using GeneMapper^® ^software (Applied Biosystems Inc., Warrington, UK). Diversity statistics were calculated according to [45] using the Excel microsatellite toolkit [46]. The unbiased estimator of Wright's inbreeding coefficient, F*_IS_*, was calculated using the FSTAT v. 2.9.3 software [47].

### Illumina genotyping

The entire genotyping procedure was performed as recommended in the Goldengate Genotyping Assay for VeraCode Manual (Illumina VC-901-1001). All reagents, unless stated otherwise were provided by Illumina. The sample VBP was scanned immediately using default settings in the VeraScan software on the BeadXpress Reader System.

### Data extraction and interpretation

Genotypes were scored visually using Illumina BeadStudio data analysis software (v 3.1) package. Each SNP was scored separately and clusters determined automatically or manually into the three expected groups (AA, AB and BB).

### Preliminary data analysis

Brennan *et al. *[14] detected 43 progeny thought to be selfs among the original 125 progeny of the SCRI 9328 population by a cluster analysis of the AFLP bands segregating in the pollen parent only. This analysis was repeated for the extended population of 311 lines, using the SNP markers that segregated in the pollen parent only. A simple matching coefficient was used as a measure of similarity, and a dendrogram was constructed using group average cluster analysis. For comparison, cluster analysis was also carried out based on the SNP markers that segregated in the seed parent only. The same analysis was carried out on the MP7 progeny. All cluster analyses were performed using Genstat for Windows 12 [48].

### Genetic mapping

Linkage maps of the segregating SNPs and SSRs were estimated for both the reference mapping population SCRI 9328 and also for the second MP7 population separately, using the JoinMap 3 software [49] and the Kosambi mapping function. Heterogeneity between recombination frequencies in the two populations was examined using the chi-squared test in JoinMap 3.

### QTL analysis of the SNP theta scores

The Illumina data consists of two intensity values (X, Y) for each SNP, measuring the intensities of the fluorescent dyes associated with the two alleles of the SNP. After normalisation, the intensities are transformed to a combined SNP intensity R = (X+Y) and an intensity ratio theta = (2/π)*arctan(Y/X) [50]. Individuals are classified as genotypes AA, AB or BB at each SNP depending on the SNP theta score.

All of the 384 SNPs were expected to segregate in population SCRI 9328, but as reported, about half were not identified as segregating by the BeadStudio software. Another approach was to analyse the theta scores as quantitative traits, regarding them as being comprised of genetic information plus measurement error. Each trait was thus analysed by QTL interval mapping using the software MapQTL 5.0 [51]. Genstat 12 was also used to carry out regressions of the theta scores on the marker data and to estimate the percentage of the variance explained.

## Authors' contributions

JR helped conceive the study and coordinated the molecular work and mapping analysis. PH helped conceive the study, provided advice on the experimental design and molecular biology, and facilitated the 2GS procedures. MB and LC provided bioinformatics support for the 2GS data. CH analysed the mapping data. CB and JAM provided sequencing and genotyping support. RB helped conceive the study and provided appropriate plant material. SG collected plant samples for analysis. LJ performed the molecular work. JR, PH and RB drafted the manuscript, which all authors read and approved.

## Supplementary Material

Additional File 1 **Figure S1 - Distribution of GO annotation categories (blue bars) of blackcurrant ESTs based upon closest derived homologies to *Arabidopsis *predicted peptide sequences**. These are compared to distribution of GO annotations from the whole *Arabidopsis *genome (red bars). Click here for file
