# Supplementary material for: Identification, utilisation and mapping of novel transcriptome-based markers from blackcurrant (Ribes nigrum)
Source: BMC Plant Biol. 2011 Oct 28;11:147. doi: 10.1186/1471-2229-11-147 (PMC3217869; doi:10.1186/1471-2229-11-147)
Supplement: Supplementary file 1 [file 1471-2229-11-147-S1.DOCX]

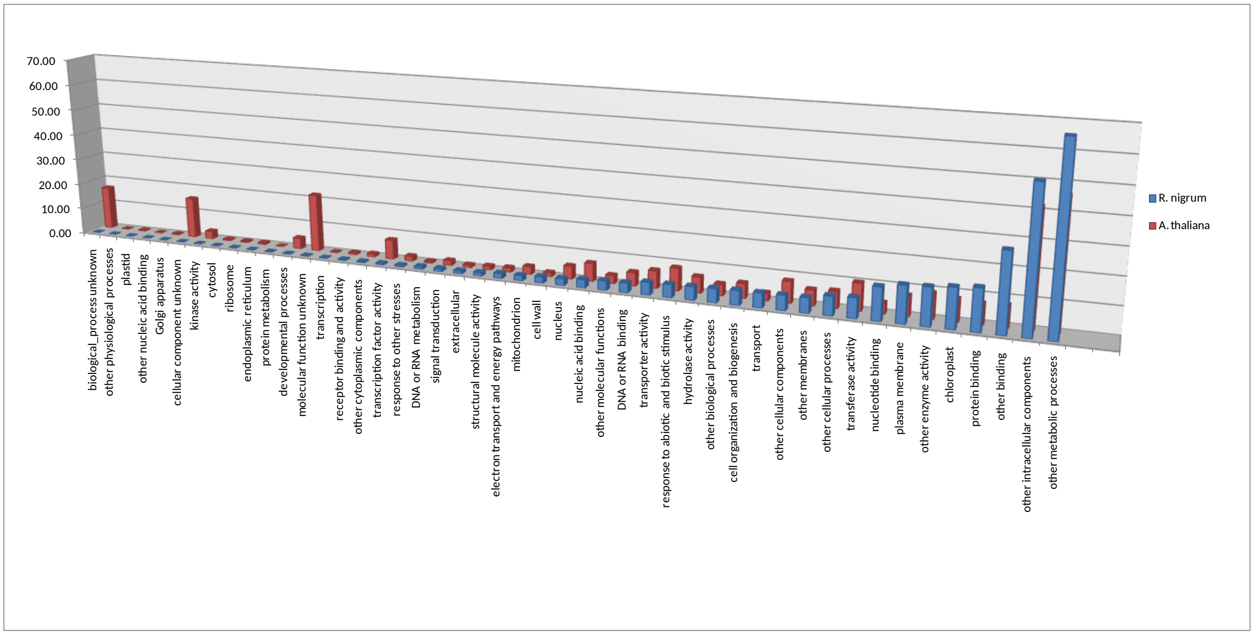

70.00
60.00
50.00
40.00
30.00
20.00
10.00
0.00
R. nigrum
A. thaliana
biological_process unknown
other physiological processes
plastid
other nucleic acid binding
Golgi apparatus
cellular component unknown
kinase activity
cytosol
ribosome
endoplasmic reticulum
protein metabolism
developmental processes
molecular function unknown
transcription
receptor binding and activity
other cytoplasmic components
transcription factor activity
response to other stresses
DNA or RNA metabolism
signal transduction
extracellular
structural molecule activity
electron transport and energy pathways
mitochondrion
cell wall
nucleus
nucleic acid binding
other molecular functions
DNA or RNA binding
transporter activity
response to abiotic and biotic stimulus
hydrolase activity
other biological processes
cell organization and biogenesis
transport
other cellular components
other membranes
other cellular processes
transferase activity
nucleotide binding
plasma membrane
other enzyme activity
chloroplast
protein binding
other binding
other intracellular components
other metabolic processes
